# Acetaminophen-induced liver injury: Molecular mechanism and treatments from natural products

**DOI:** 10.3389/fphar.2023.1122632

**Published:** 2023-03-27

**Authors:** Jiaqing Liao, Qiuxia Lu, Zhiqi Li, Jintao Li, Qi Zhao, Jian Li

**Affiliations:** ^1^ Engineering Research Center of Sichuan-Tibet Traditional Medicinal Plant, Chengdu University, Chengdu, China; ^2^ School of Pharmacy, Chengdu University, Chengdu, China; ^3^ School of Food and Biological Engineering, Chengdu University, Chengdu, China; ^4^ School of Basic Medical Sciences, Chengdu University, Chengdu, China

**Keywords:** acetaminophen, liver injury, natural products, oxidative stress, Nrf2

## Abstract

Acetaminophen (APAP) is a widely used analgesic and antipyretic over-the-counter medicine worldwide. Hepatotoxicity caused by APAP overdose is one of the leading causes of acute liver failure (ALF) in the US and in some parts of Europe, limiting its clinical application. Excessive APAP metabolism depletes glutathione and increases N-acetyl-p-benzoquinoneimide (NAPQI) levels, leading to oxidative stress, DNA damage, and cell necrosis in the liver, which in turn leads to liver damage. Studies have shown that natural products such as polyphenols, terpenes, anthraquinones, and sulforaphane can activate the hepatocyte antioxidant defense system with Nrf2 as the core player, reduce oxidative stress damage, and protect the liver. As the key enzyme metabolizing APAP into NAPQI, cytochrome P450 enzymes are also considered to be intriguing target for the treatment of APAP-induced liver injury. Here, we systematically review the hepatoprotective activity and molecular mechanisms of the natural products that are found to counteract the hepatotoxicity caused by APAP, providing reference information for future preclinical and clinical trials of such natural products.

## 1 Introduction

Acetaminophen (APAP), also called paracetamol, is one of the most widely used analgesic and antipyretic over-the-counter drugs in the world (Lee, 2008; McCracken, 2015). According to the statistics, more than 60 million Americans take APAP every week. It is also worth noting that APAP is used in combination with other drugs, particularly opioids and diphenhydramine, without public awareness (Athersuch et al., 2018; Jasani et al., 2018; Rajaram and Subramanian, 2018). Although APAP is considered safe under therapeutic doses, its overdose can induce severe liver toxicity and even death (Agrawal and Khazaeni, 2022). The hepatotoxicity resulting from an overdose of APAP is the leading cause of acute liver failure (ALF) in the United States and in some parts of Europe, accounting for more than 50% of ALF cases in these regions (Yoon et al., 2016). In severe circumstances, liver transplantation is the only option that might possibly save the patient’s life. APAP-induced liver injury is emerging as a public health issue (Yang et al., 2022a).

**FIGURE 1:**
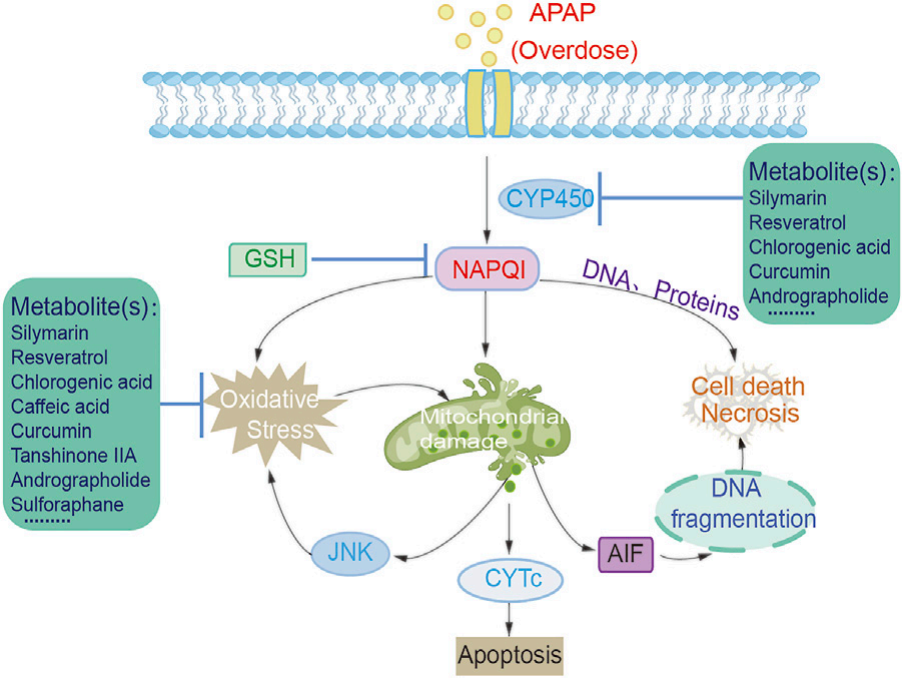
Metabolites protect against acetaminophen-induced hepatotoxicity.

The precise molecular mechanism of APAP-induced liver injury has not been fully elucidated yet. Under the therapeutic concentrations, approximately 60%–90% of APAP is metabolized in the liver by glucuronidation and sulfation, with a small part (approximately 5%–15%) being metabolized by the cytochrome P450 pathway (CYP450) (Kalsi et al., 2011; Marto et al., 2021). Probably due to the binding preference of APAP to the active site of each P450 isomer, it is oxidized *via* two pathways to form the toxic intermediate N-acetyl-p-benzoquinone imine (NAPQI) and the non-toxic catechol metabolite 3-hydroxy-APAP (3-OH-APAP) (Chen et al., 1998). Simultaneous quantification of these two oxidized metabolites by electrochemical HPLC assay demonstrated that human P450 2E1 selectively oxidizes APAP to NAPQI (determined as glutathione conjugate, GS-APAP), whereas human P450 2A6 selectively oxidizes APAP to 3-OH-APAP (Chen et al., 1998). Under the overdose condition, more APAP is converted to NAPQI by cytochrome P450 enzymes (Figure 1). Chen et al. (1998) NAPQI oxidizes the thiol groups of proteins and generates reactive oxygen species (ROS) (Iorga et al., 2017). Both NAPQI and ROS cause mitochondrial DNA damage, activation of the JNK signaling pathway, which further amplifies the mitochondrial ROS production, and causes the mitochondrial permeability transition (MPT) pore to open (Iorga et al., 2017). Glutathione conjugates with the generated NAPQI to harmless thiolate and cysteine compounds, which will be eliminated by the kidney. Overdose of APAP depletes glutathione reservoir, leading to the rise of NAPQI level. NAPQI will then bind to the cellular macromolecules, including proteins, lipids, and nucleic acids, resulting in centrilobular liver injury and hepatocyte death (McGill et al., 2012; More et al., 2017; Ramachandran et al., 2018; Guengerich, 2020). At earlier time, the cell death resulted from APAP toxicity has been suggested to be apoptosis (Ray et al., 1996; El-Hassan et al., 2003), necrosis (Iorga and Dara, 2019; Jaeschke et al., 2019). However, no definitive conclusion could be reached with the current evidence. Ferroptosis has also been indicated to be involved in APAP hepatotoxicity (Yamada et al., 2020), which is not supported by the data obtained under pathophysiologically relevant condition (Jaeschke et al., 2021; Adelusi et al., 2022). More recently, several studies suggested that pyroptosis is the type of cell death after APAP overdose (Wang et al., 2021; Liu et al., 2022; Rousta et al., 2022).

Currently, N-acetylcysteine (NAC) is the only U.S. Food and Drug Administration approved treatment for APAP induced liver injury (Andrade et al., 2019). However, because of the narrow therapeutic window for acute liver injury and the side effects of NAC, its clinical application has great limitations (Du et al., 2016). Inhibitors targeting the enzymatic activity of P450, such as 4-methylpyrazole (Fomepizole), are evaluated *in vitro* and *in vivo* as possible alternative treatments for APAP induced liver injury (Akakpo et al., 2018; Akakpo et al., 2019; Kang et al., 2020; Akakpo et al., 2022; Kaiser and Dart, 2022). Natural products have long been considered important sources of novel medicines and therapeutics. A number of natural products, such as triterpenoid saponins (Xu et al., 2018), schisandra lignans (Zhu et al., 2019), polysaccharides (Wang et al., 2018), iridoids (Shao et al., 2017), flavonoids and quinones (Darvin et al., 2018), have been shown to have protective activity against APAP-induced hepatotoxicity. In this review, we summarized the most recent research progress on natural product-derived ingredients, which have beneficial effects on the liver injury caused by APAP. The information from this review could serve as a reference for the further development of natural product-based treatments for APAP-induced liver injury.

## 2 Effects and mechanism of natural products in acetaminophen-induced hepatotoxicity

**FIGURE 2:**
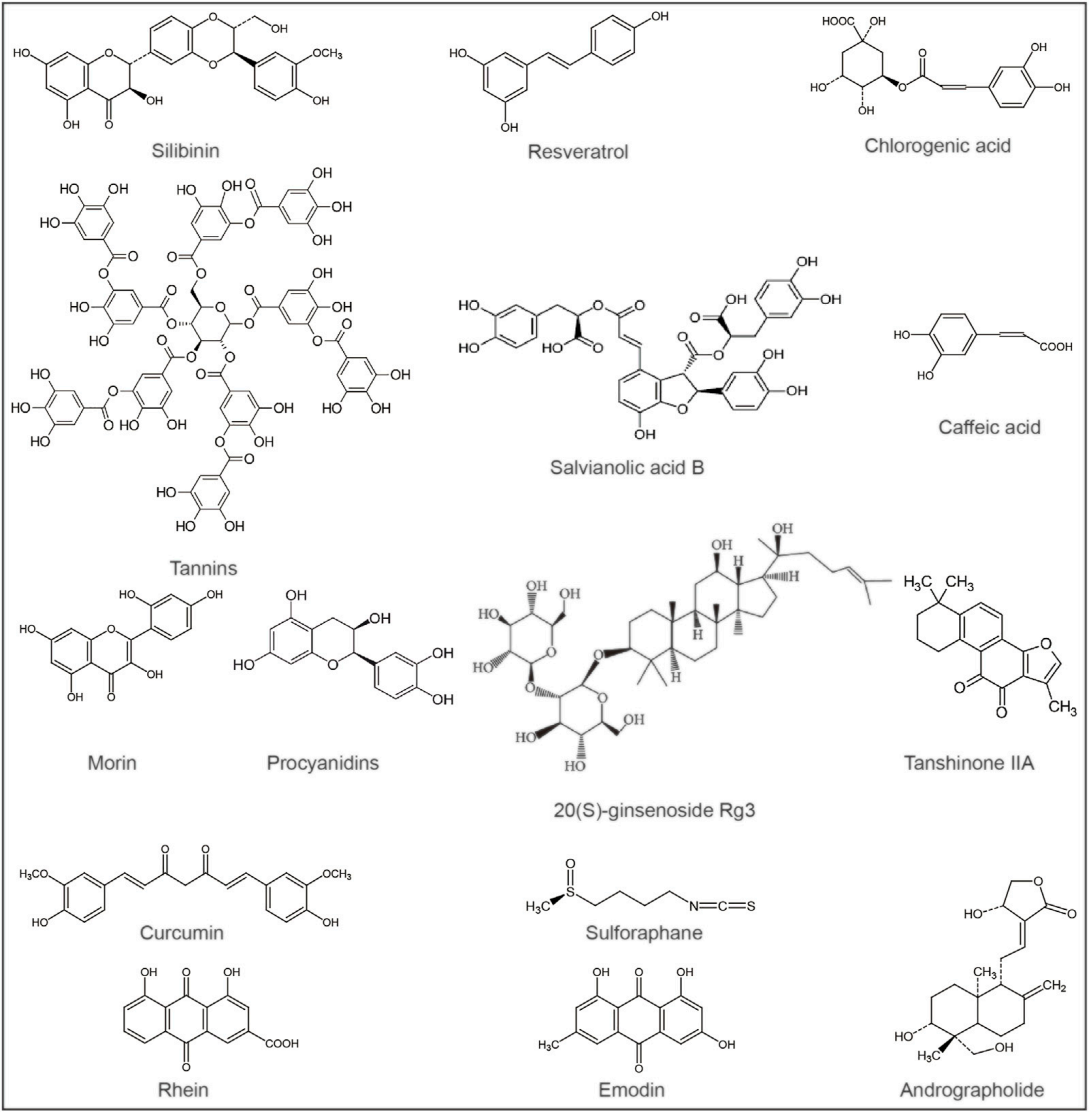
Chemical structures of potential metabolites for the treatment of acetaminophen-induced liver injury.

A number of phytochemicals have been identified to have hepatoprotective activity, such as silymarin for the treatment of liver poisoning, chronic hepatitis and cirrhosis (Abenavoli et al., 2018), resveratrol and curcumin for the relief of liver damage, etc., (McGill et al., 2015; Khan et al., 2019) (Table 1). Liver-protecting natural products often have diverse activities, including antioxidant, anti-inflammatory, immunomodulatory, and antiviral effects (Kim et al., 2015; Ilyas et al., 2016; Abdullah et al., 2017; Sharifi-Rigi et al., 2019). These compounds have the effect of alleviating APAP-induced liver injury and can be further developed as antioxidants or hepatoprotective agents (Figure 2).

**TABLE 1:** Metabolites relieves acetaminophen-induced liver injury.

Categories	Metabolites	Species name	Model	Does or test concentrations	Minimal active does	Positive/negative control	Efficacy	References
Polyphenols	Silymarin	*Silybum marianum* (L.) Gaertn. [Asteraceae]	Balb/c mice APAP (300 mg/kg b.wt. i.p.)6 h, 12 h and 24 h	pretreated 100 mg/kg/d (b.wt. per os) 3d	100 mg/kg	silymarin and 25% xanthan gum i.g.	↑HO-1, ↓superoxide, GSSG, p-JNK, ROS	Papackova et al. (2018)
			Domestic pigeons (Columba livia)APAP (3,000 mg/kg PO q24 h) 0 h, 12 h, 24 h, 48 h, 72 h	35 mg/kg, starting at 12 h after APAP, silymarin treatment q12 h for 0 h, 12 h, 24 h, 48 h, 72 h.	35 mg/kg	tap water;	↓mortality	Ihedioha et al. (2020)
						PBS(37°C)		
			Male C57/BL6 mice, APAP (350 mg/kg i.p.)6 h, 12 h	oral 35/50/65 mg/kg (dissolved in 4% HP-β-CD (w/v)) 21d	35 mg/kg	4% HP-β-CD (w/v); saline	↓CYP2E1, NAPQI	Yang et al. (2022b)
	Resveratrol	*Vitis vinifera* L. [Vitaceae], *Vaccinium myrtillus* L. [Ericaceae], *Rubus idaeus* L. [Rosaceae], *Morus alba* L. [Moraceae], *Arachis hypogaea* L. [Fabaceae]	Male C57BL/6 mice, APAP (400 mg/kg i.p.) 6 h	oral, pretreated 25/50/100 mg/kg, 7 times interval 12 h	100 mg/kg	0.5% CMC-Na; saline	↓JNK, p53, CYP2E1, CYP3A11 and CYP1A2, ↑SIRT1, cyclin D1, CDK4, PCNA	Wang et al. (2015)
	Tannins	*Osyris lanceolata* Hochst. and Steud. [Santalaceae]	Kunming mice, APAP (400 mg/kg, i.p.) 12 h	oral Tannins (25/50mg/kg), silymarin (100mg/kg) 3d	25 mg/kg	silymarin (100 mg/kg); saline	↑Nrf2, HO-1,bcl-2,↓IL-1β, TNF-α, c-fos, c-jun, NF-κB (p65), caspase-3, bax	Zhang et al. (2017)
	Salvianolic acid B	*Salvia miltiorrhiza Bunge* [Lamiaceae]	Male Kunming mice, APAP (300 mg/kg,i.g.24 h; HepG2 cells, APAP (10 mM) 24 h	pretreated,25/50 mg/kg,3d; 0.5, 2, 8 μmol/L, 6 h	25 mg/kg; 8 μmol/L	double-distilled water(37 °C)	↑PI3K, PKC,Nrf2, HO-1, GCLC	Lin et al. (2015)
	Chlorogenic acid	*Phyllostachys edulis* (Carrière) J.Houz. [Poaceae]	Male ICR mice, oral ,APAP (300 mg/kg) 4 h	pretreated, oral,5/10/20/40 mg/kg, 7 d	20 mg/kg		↑MAPK, GCLC, Trx1/2,TrxR1↓ERK1/2,JNK, p38,ASK1, cRaf, MEK1/2, MKK4, MKK3/6, caspase-3/7,	Ji et al. (2013)
			ICR and C57BL/6 mice, oral, APAP (300 mg/kg)6 h; L-02cell, APAP (10/7.5 mM) 4/8/18/24/36/48h	pretreated, oral,20/40 mg/kg,6d; pretreated, 25/50 μM,15/30 min	20 mg/kg; 25 μM		↑Nrf2,HO-1,NQO1,p-ERK1/2, ↓PP2A-A, PP5,ROS	Wei et al. (2018)
			Male C57BL/6 mice, oral APAP (300 mg/kg) 6 h	oral, 40 mg/kg(1 h after APAP) 5h	40 mg/kg		↑Nrf2,Lon,↓HSP60,HMGB1, IL-1β,COX2,TNFα,iNOS, NRF1	Hu et al. (2020)
			Male Kunming mice, APAP (300 mg/kg, i.g.) 24 h; HepG2 cell	20/40 mg/kg (i.g.), 14 d; pretreated, 12.5/25/50uM, 15 min	20 mg/kg; 25uM	Ammonium glycyrrhizinate (AG) (200 mg/kg), 14 d; 0.9% saline (i.g.)	↑PINK1, Parki, LC3II/LC3I, ↓p62, Tom20	Hu et al. (2022)
	Caffeic acid	*Ilex paraguariensis* A.St.-Hil. [Aquifoliaceae]	Male ICR mice, oral, APAP (400 mg/kg)4 h; L-02/HepG2 cells, APAP (7.5/10 mM) 4/8/18/36 h	oral,10/30 mg/kg, 7d; pretreated,10/25/50mM, 15 min	30 mg/kg; 25 μM		↑Keap1-Nrf2,Nrf2,HO-1, NQO1,↓ROS,Keap1, CYP2E1, CYP3A4	Pang et al. (2016)
	Morin	*Maclura pomifera* (Raf.) C.K.Schneid. [Moraceae], *Maclura tinctoria* (L.) D.Don ex G.Don [Moraceae], *Psidium guajava* L. [Myrtaceae]	Male Wistar rats, oral APAP (1 g/kg/d, 0.5 %CMC) 28d	oral 30 mg/kg/d, 0.5% CMC, 28 d	30 mg/kg	0.5% CMC	↑Nrf2,HO-1,NQO1, Nrf2 nuclear transfer and ARE-Nrf2 affinity,↓PHLPP2, pFyn,GSK3β,HMGB1,caspase-12, Nrf2 ubiquitination	Rizvi et al. (2015b)
	Procyanidins	*Prunus amygdalus* Batsch [Rosaceae]	Male Balb/c mice, APAP (300 mg/kg,i.p.)8 h; HepG2	oral, Procyanidins 1/10 mg/kg, silymarin (50 mg/kg), three times per week; 10/25/50 μg/mL, 12 h	1 mg/kg; 25 μg/mL	silymarin (50 mg/kg); Sulforaphane (25 μM);	↑Nrf2/ARE, ERK, PI3K/Akt, NQO1, GPX, SOD	Truong et al. (2014)
						50% polyglycol;		
						0.1%DMSO		
	Curcumin	*Curcuma longa* L. [Zingiberaceae]	Male B6C3F1 mice, APAP (400 mg/kg, i.p.) 24 h	17 mg/kg/day (p.o.) 12 d	17 mg/kg		↓Bax, caspase-3, p53,↑Bcl-XL	Bulku et al. (2012)
			Male BALB/c mice, APAP 300 mg/kg (i.p.) 16 h	10/20 mg/kg (i.p.) 2 h	10 mg/kg	PBS; 1% CMC	↑Bcl-2/Bax,↓liver necrosis	Li et al. (2013)
			Male mice, fed, APAP 400 mg/kg, 24 h	fed, 200/600 mg/kg, 24 h	200 mg/kg	corn oil;	↓necrosis, IL-12, IL-18	Somanawat et al. (2013)
						distilled water		
			Male CD1 mice, APAP (350 mg/kg bw i.p.) 14 h	35/50/100 mg/kg, bw, 90min	35 mg/kg	0.05% CMC	attenuated the decrease in oxygen consumption, membrane potential, ATP synthesis, aconitase, respiratory complexes I, III, IV	Granados-Castro et al. (2016)
Terpenes	Ginsenosides	*Panax ginseng* C.A.Mey. [Araliaceae]	Male ICR mice, oral APAP (200-500 mg/kg) 18 h; H4IIE cells	oral, 10/30/100/300/500 mg/kg/day, 1/5weeks; 0.1/0.5/1 mg/ml, 12 h	30 mg/kg; 0.1 mg/ml	40% PEG400; distilled water	↓LD50, P450 2E1, CYP2E1 ↑GSTA2, Nrf2, C/EBPβ, C/EBPb, GSTA2	Gum and Cho (2013b)
	Tanshinone IIA	*Salvia miltiorrhiza Bunge* [Lamiaceae]	male C57BL/6J mic, APAP (i.p.,300 mg/kg) 24 h; HepG2 cells	oral,10/30 mg/kg,4 d; 10 μM, 24 h	30 mg/kg; 10 μM	0.5% CMC-Na (20 ml/kg)	↑Nrf2, GCLC, NQO1, HO-1	Wang et al. (2016)
	Andrographolide	*Andrographis paniculata* (Burm.f.) Nees [Acanthaceae]	C57BL/6 mice, APAP (300 mg/kg, i.g.)6weeks	20/40 mg/kg (i.g.), 4 weeks	20 mg/kg	4% methyl-cyclodextrin	↑Nrf2,GCL, NQO1, HO-1, P62,↓Keap1, ROS	Yan et al. (2018)
Anthraquinones	Rhein	*Rheum palmatum* L. [Polygonaceae]	Male Sprague–Dawley rats, 2.5 g/kg APAP (i.g.) 48 h	10/20/40 mg/kg (i.g.) 48 h	10 mg/kg	5%CMC-Na;	↓ROS, NO, MDA, ↑GSH	Zhao et al. (2011)
						Saline+0.2% gum (i.g.)		
	Emodin	*Rheum palmatum* L. [Polygonaceae]	Female Sprague-Dawley albino rats, APAP (2 g/kg, po) 48 h	20, 30 and 40 mg/kg (po) 24 h	30 mg/kg	silymarin (50 mg/kg, po); NaHCO3; hot distilled water	↓MDA, SALP, LDH, LFTs, ↑GSH	Bhadauria (2010)
			Male C57BL/6 mice, APAP (300 mg/kg,i.p.) 24 h	pretreated, oral 15/30 mg/kg, 5 d	30 mg/kg	40% PEG; saline	↑Nrf2,NQO1, HO-1,↓NLRP3,IL-1β, IL-6, TNF-α,IFN-α, cGAS, STING,CYP2E1	Shen et al. (2022)
Sulfur-containing NPs	Sulforaphane	*Brassica oleracea* L*.*	Male, C57BL/6 mice APAP (300 mg/kg,i.p.)6 h; Primary hepatocytes, APAP (15 mM) 14 h	pretreated, oral 5 mg/kg,30min; pretreated SFN (10 μM) 6 h	5 mg/kg; 10 μM	PBS	↑Nrf2,Gclc, Gclm, Cu/Zn SOD, HO-1,↓ROS,4-HNE	Noh et al. (2015)
			Male Sprague-Dawley rats, oral APAP 1 g/kg (3 h after Sulforaphane), 24 h	oral 500 μg/kg/d, 3 d	500 μg/kg	water; hot saline	↓neopterin, CRP, cellular inflammation, liver damag, protect normal hepatic architecture	Dokumacioglu et al. (2017)

### 2.1 Polyphenols

Silymarin, the flavonoid extract from the *Silybum marianum* (L.) Gaertn. [Asteraceae] plant, has been used to prevent various liver diseases (Polyak et al., 2010). Silymarin has been reported to act as an antioxidant by reducing free radical production and lipid peroxidation, function as a toxin blocker by inhibiting the binding of toxins to the hepatocyte cell membrane receptors (Abenavoli et al., 2010), and reduce the superoxide and peroxynitrite content by its scavenger activity (Papackova et al., 2018). Recent research has shown that silymarin reduces acute toxic liver injury caused by APAP by increasing hepatocyte proliferation, decreasing CYP2E1 activity and expression, and decreasing the production of toxic metabolites (Elsayed Elgarawany et al., 2020; Ihedioha et al., 2020; Yang et al., 2022b). Silibinin is the major active component of silymarin. The nanoparticles of silibinin exhibited antioxidant effects against intracellular oxidative stress by upregulating the Nrf2/ARE pathway, reducing ROS, modulating antioxidant enzyme responsiveness, and inhibiting downstream pathways of mitochondrial damage (Ding et al., 2022). The above findings suggest that silymarin has the potential to be further developed as an antioxidant against APAP.

Resveratrol, a non-flavonoid phenolic substance, has been shown to have a hepatoprotective effect by attenuating oxidative stress in the liver (Das, 2011; Dalaklioglu et al., 2013; Ahmad and Ahmad, 2014). It was found to be an irreversible inhibitor of CYP3A4 and a non-competitive reversible inhibitor of CYP2E1 (Piver et al., 2001; Wang et al., 2015). It also inhibits the activity of CYP3A11 and CYP1A2, preventing the bioactivation of APAP to the toxic metabolite NAPQI (Piver et al., 2001; Wang et al., 2015). Resveratrol treatment has been shown to reduce oxidative stress in mouse tissues by increasing the expression of antioxidants and phase II enzymes in response to the stress (Rubiolo et al., 2008; Cerný et al., 2009; Wong et al., 2009). Interestingly, studies have shown that the antioxidant activity of this compound has a circadian rhythm, having antioxidant properties when it is in the dark but pro-oxidant properties when it is in the light (Gadacha et al., 2009). SIRT6 downregulation inhibits Nrf2 activation, whereas sirtuin 6 (SIRT6) upregulation reduces oxidative stress-associated DNA damage and promotes hepatocyte proliferation, thereby preventing APAP hepatotoxicity (Zhou et al., 2021). Resveratrol, a potent SIRT1 activator, promotes cell survival by regulating SIRT1-dependent p53 deacetylation (Howitz et al., 2003; Kim et al., 2011). Another study showed that resveratrol induced SIRT1 expression and the expression of cyclin D1, cyclin-dependent kinase 4 (CDK4) and proliferating cell nuclear antigen (PCNA) to promote liver regeneration, thereby preventing acetaminophen-induced hepatotoxicity (Wang et al., 2015). In addition, resveratrol has been shown to protect against APAP-induced liver injury when administered therapeutically. It exerts a protective effect by scavenging peroxynitrite and preventing the release of AIF and EndoG from mitochondria and subsequent nuclear DNA breakage (Du et al., 2015). The above studies suggest that resveratrol may be an effective option for the treatment of APAP overdose.

Tannins is well-known for its anti-oxidative stress activity. The ability of taninins to protect the liver from damage caused by APAP has also been looked into. Tannins significantly reduced the phase I and phase II enzyme activities in mouse liver tissues (Krajka-Kuźniak and Baer-Dubowska, 2003). Tannic acid exerts anti-apoptotic activity by down-regulating the caspase-3, Bax, and up-regulating Bcl-2, and activates the antioxidant defense system by up-regulating Nrf2 and HO-1 (Zhang et al., 2017).

Salvianolic acid B, a rosmarinic acid dimer, is an active component of *Salvia miltiorrhiza Bunge* [Lamiaceae], and its reduction of drug-induced liver injury is associated with its antioxidant activity (Gao et al., 2012; Lin et al., 2015). In HepG2 cells, salvianolic acid B inhibited the expression of CYP3A4 and CYP1A2, induced the expression of GST (Wang et al., 2011). Also, salvianolic acid B pretreatment induces Nrf2 and phase II enzymes by activating phosphoinositide 3-kinase (PI3K) and protein kinase C (PKC) pathways, thereby preventing acetaminophen-induced hepatotoxicity in mice (Lin et al., 2015). The above studies suggest that the hepatoprotective activity of salvianolic acid B against APAP is achieved by inhibiting cytochrome P450 enzymes and/or synergizing phase II metabolic enzymes.

Chlorogenic acid, a phenolic compound with various biological activities, ameliorates liver injury in an experimentally induced model of oxidative stress (Pang et al., 2015). Chlorogenic acid is a P450 enzyme inhibitor, which can decrease the expression of CYP2E1 and CYP1A2 (Pang et al., 2015). Chlorogenic acid inhibited APAP-induced activation of caspase-3 and caspase-7, JNK, ERK1/2, and upstream molecular signaling of p38 MAPKs in animals, including apoptosis signal-regulated kinase 1 (ASK1), c-Raf, and Mitogen-activated protein kinases MEK1/2, MKK3/6 and MKK4 (Ji et al., 2013), thereby inhibiting apoptosis. Chlorogenic acid inhibits the binding of Nrf2 to its repressor protein Keap1 to activate the Nrf2 antioxidant signaling pathway, thereby preventing APAP-induced hepatotoxicity (Wei et al., 2018). Apart from that, the activation of Nrf2 by chlorogenic acid restores mitochondrial ion protein homologue (Lon) protein expression and reduces mitochondrial HSP60 release, attenuating APAP-induced inflammatory liver injury (Hu et al., 2020). Studies have shown that the hepatotoxicity of APAP can lead to mitochondrial dysfunction and affect PINK1-mediated mitosis. Chlorogenic acid stabilizes cell function by eliminating mitochondrial damage, increases PINK1-dependent mitosis, inhibits apoptosis of liver cells, and prevents APAP hepatotoxicity (Hu et al., 2022). Currently, chlorogenic acid is considered a promising hepatic detoxifier for APAP.

Caffeic acid is a common phenolic chemical found in a wide variety of plants. It may protect L02 cells from acetaminophen-induced damage by activating the Keap1-Nrf2 antioxidant defense mechanism. Studies have shown that caffeic acid can inhibit the expression of Keap1, reduce the stabilization of the Keap1 and Nrf2 complex, thereby activate Nrf2 and upregulate the expression of downstream antioxidant enzymes NQO1 and HO-1 (Pang et al., 2016).

Morin, a type of flavonol, is obtained from the wood of the Morus alba plant and has a variety of biological activities, including antioxidant, hypoglycemic, and liver protection. Studies have shown that it can resist the toxicity of APAP to hepatocytes by activating Nrf2. Specifically, the inhibition of Nrf2 ubiquitination increases nuclear Nrf2 retention and ARE-Nrf2 binding affinity (Rizvi et al., 2015a).

Procyanidins from almonds (a subclass of procyanidins), demonstrated protective efficacy against APAP-induced hepatotoxicity in HepG2 cells and mice (Truong et al., 2014). The fundamental mechanism is the activation of phase II detoxification enzymes or antioxidase controlled by Nrf2/ARE, including the expression of NAD(P)H quinone dehydrogenase 1 (NQO1), GPX, and superoxide dismutase (SOD) (Truong et al., 2014). At present, procyanidins are mainly used to alleviate AILI through antioxidants, and whether there are other effects needs further research.

Curcumin, a yellow phenolic pigment extracted from the rhizome of *Curcuma longa* L. [Zingiberaceae], belongs to the diarylheptane class of metabolites, and is known for its ability to treat a variety of human diseases (Shishodia et al., 2005). According to reports, curcumin is a P450 inhibitor that inhibits CYP2C9, CYP1A2, CYP2D6, CYP2B6 and CYP3A4, with particularly low IC50 values for CYP2C9 (Appiah-Opong et al., 2007). In the system of Ad-P450 cells, it inhibits five P450 enzymes in a concentration-dependent manner (Sasaki et al., 2017). Curcumin inhibits APAP-induced hepatocyte apoptosis by reducing the expression of pro-apoptotic genes Bax and caspase-3, inducing anti-apoptotic genes like Bcl-x1, and increasing the ratio of Bcl2/Bax (Bulku et al., 2012; Li et al., 2013). Curcumin prevents APAP-induced hepatitis by reducing oxidative stress, decreasing liver inflammation, restoring GSH and improving liver histopathology (Somanawat et al., 2013). Furthermore, its protective effect in APAP-induced hepatotoxicity was linked to reduced mitochondrial dysfunction, oxygen consumption, and membrane potential (Granados-Castro et al., 2016). At the same time, a series of studies reported that curcumin also exerts hepatoprotective activity by activating the Nrf2 signaling pathway and regulating ARE-driven antioxidant genes (Lu et al., 2015; Liu et al., 2017; Krupa et al., 2019).

### 2.2 Terpenes

Ginsenosides are the main steroid chemicals found in ginseng roots, extracted from *Panax ginseng* C.A.Mey. [Araliaceae]. Ginsenoside Rg3 promotes the expression of multidrug resistance proteins (MRP) 1 and 3, activates Nrf2-mediated antioxidant gene expression, participates in detoxification, and reduces liver cytotoxicity (Gum and Cho, 2013a). Meanwhile, ginsenoside Rg3 significantly increased glutathione S-transferase α2 (GSTA2) protein expressionand activated the transcription of GSTA2 downstream of multiple cellular signaling pathways, including protein kinase A (PKA), PI3K and JNK (Gum and Cho, 2013b). Other studies have also shown that it exerts hepatoprotective effects through its anti-oxidant activity. For example, ginsenosides has been shown to increase GPX, SOD and catalase (CAT) activity and restore GSH levels, while inhibiting ERK and JNK MAPK pathways (Park et al., 2012; Li et al., 2014). Therefore, ginsenosides mainly reduce the hepatotoxicity of APAP by inhibiting oxidative stress.

Tanshinone IIA is isolated from *Salvia miltiorrhiza Bunge* [Lamiaceae] as a diterpene quinone (Fu et al., 2007). It can inhibit various CYP substrates and CYP isomers (Wang et al., 2010). According to the results from *in vitro* and *in vivo* studies, tanshinone IIA pretreatment protects the liver from APAP-induced liver injury by activating Nrf2 and increasing the mRNA and protein levels of the Nrf2 target genes glutamate-cysteine ligase catalytic subunit (GCLC), NQO1, and HO-1 (Wang et al., 2016).

Andrographolide, a ladanditerpene extracted from Andrographis paniculata, is a MAPK/Nrf2 pathway activator (Yang et al., 2017). Studies have shown that it inhibits the mRNA and protein expression of CYP1A2, CYP2D6 and CYP3A4 (Ooi et al., 2011). Andrographolide inhibits CYP3A4 activity by binding and antagonizing PXR function, and is a potential CYP3A4 inhibitor that may have clinical significance (Ooi et al., 2011). In addition, andrographolide has a protective effect on APAP-induced hepatotoxicity both *in vivo* and *in vitro* (Yan et al., 2018). Mechanistically, it activates Nrf2 and its nuclear translocation, thereby enhancing the expression of downstream antioxidant genes to relieve oxidative stress.

### 2.3 Anthraquinones

Rhein, an anthraquinone derivative of *Rheum palmatum* L. [Polygonaceae], induces apoptosis through a caspase-dependent pathway (Shi et al., 2008). Rhein has been shown to reduce APAP-induced oxidative damage to the liver cells (Zhao et al., 2011). Compared with the rats treated with APAP alone, rhein treated animals demonstrated significant reduction of the biochemical indicators of liver injury, including aspartate aminotransferase (AST), alanine aminotransferase (ALT), urea nitrogen (UREA), creatinine (CREA), nitric oxide (NO), and malondialdehyde (MDA). The glutathione (GSH) content was significantly restored, and the histopathological damage in the liver was also significantly improved after rhein treatment. However, its mechanism of action needs to be further studied (Zhao et al., 2011). It has been shown that rhein inhibits CYP2C9, CYP1A2, CYP2E1, CYP2D6 and CYP3A enzymes in rat liver (Tang et al., 2009). It is possible that rhein relieves APAP-induced liver injury through inhibiting the activity of P450 enzymes.

Emodin is an emodin compound isolated from *Rheum palmatum* L. [Polygonaceae] plants. Emodin and aloe-emodin are isomers with inhibitory effects on CYP1B1 activity (Meng et al., 2022). The conformational relationship indicates that aloe-emodin is more effective due to the different positions of the hydroxyl groups (Meng et al., 2022). Studies have found that emodin has protective effects on the APAP-induced acute liver injury in rats (Bhadauria, 2010). Emodin pretreatment significantly decreased ALT, AST and alkaline phosphatase (ALP) levels; increased albumin (ALB) levels; attenuated SOD and GSH depletion and MDA accumulation. The upregulation of the antioxidant enzymes, including Nrf2, HO-1 and NQO1, eventually leads to the relief of oxidative stress. To protect the liver from acetaminophen-induced inflammation and apoptosis, emodin suppresses interferon (IFN)-α, cyclic GMP-AMP synthase (cGAS), and downstream stimulators of interferon gene (STING) expression (Shen et al., 2022). Emodin also inhibits NLRP3 expression and reduces pro-inflammatory factors like interleukin-1β (IL-1β), IL-6 and TNF-α. These findings imply that emodin protects hepatocytes from APAP-induced liver damage by activating Nrf2-mediated antioxidant stress pathways, inhibiting the NLRP3 inflammasome, and downregulating the cGAS-STING signaling pathway (Shen et al., 2022). Our current understanding is that emodin can exert therapeutic effects through multiple pathways, including antioxidant, anti-inflammatory, and inhibition of CYP450.

### 2.4 Sulfur-containing natural products

Sulforaphane is a metabolite that protects the liver from the toxicity caused by conventional drugs (Noh et al., 2015; Nazmy et al., 2017). Previous research has shown that the hepatoprotective effect of sulforaphane is associated with the suppression of cytochrome P450 enzymes (McCarty, 2001). Inhibition of CYP450 enzymatic activity protects hepatocytes from the toxic metabolites of APAP. The metabolites of sulforaphane inhibit CYP2D6 activity (Vanduchova et al., 2016). Further studies showed that sulforaphane exerts hepatoprotective effects through activating the Nrf2 pathway (Hu et al., 2004). Since oxidative stress is the main cause of APAP-induced hepatotoxicity, sulforaphane protects the liver from APAP overload toxicity by activating the Nrf2 pathway and increasing the endogenous antioxidant response (Schmidt, 1984). One potential mechanism is the Nrf2-ARE pathway, which induces a phase 2 detoxification response that promotes disruption of Nrf2-Keap protein interactions, translocation of Nrf2 to the nucleus, and regulation of target gene expression through the ARE, enhancing cellular defense against oxidative damage (Hu et al., 2004; Lau et al., 2008). Antimycin-like interaction of sulforaphane with the mitochondrial respiratory chain at the complex III level generates ROS, leading to membrane lipid peroxidation and 4-hydroxynonenal production (Sharma et al., 2010). 4-Hydroxynonenal is involved in the signaling of cell proliferation and apoptosis, as well as regulating gene expression in different cell types (Cheng et al., 2001; Yang et al., 2001; Yang et al., 2002; Sharma et al., 2004; Awasthi et al., 2005; Vatsyayan et al., 2011). Interestingly, it activates defense mechanisms against oxidative stress at low concentrations, such as Nrf2 and heat shock factor 1 (Sharma et al., 2008a; Sharma et al., 2008b; Chaudhary et al., 2010), but induces apoptosis at higher supraphysiological concentrations (Awasthi et al., 2008). The study results showed that sulforaphane pretreatment significantly induced the expression of Nrf2, HO-1 and Nqo1 mRNAs and suppressed APAP-induced glutathione (GSH) depletion and lipid peroxidation (Noh et al., 2015). Therefore, sulforaphane should be cautiously developed as a treatment for APAP-induced liver injury. The combination of sulforaphane and APAP at low doses decreased intracellular ROS formation and increased the protein levels of CAT, GPx, Nrf2, NQO1, and HO-1. It indicates that sulforaphane protects against oxidative damage by APAP by enhancing cellular antioxidant activity (Vuong et al., 2019).

## 3 Conclusion

Understanding the rationale for the hepatoprotective activity of natural products could guide future drug development. Inhibition of CYP450 enzyme activity and activation of the Nrf2 signaling pathway and GSH synthesis are two intriguing targets for the treatment of acute liver injury caused by APAP. However, improving the therapeutic window of natural hepatoprotective agents, developing drug carriers with excellent properties, and reducing their toxicity remain current and future problems to be overcome. Well-designed randomized clinical studies are needed to systematically evaluate the evidence for the use of these hepatoprotective agents as treatment options for APAP-induced liver injury, to determine the timing of initiation of the therapy, and to further define the optimal treatment regimen.

## Glossary

APAP: Acetaminophen
ALF: acute liver failure
NAPQI: N-acetyl-p-benzoquinoneimide
NAC: N-acetylcysteine
AILI: APAP-induced liver injury
COX: cyclooxygenase
GPX: glutathione peroxidase
CYP450: cytochrome P450 system
ROS: reactive oxygen species
HO-1: heme oxygenase 1
iNOS: inducible nitric oxide synthase
CDK4: cyclin-dependent kinase 4
SIRT1: Sirtuin 1
TNF-α: tumor necrosis factor-α
AIF: apoptosis-inducing factor
PI3K: phosphoinositide 3-kinase
PKC: protein kinase C
ASK1: apoptosis signal-regulated kinase 1
NQO1: NAD(P)H quinone dehydrogenase 1
SOD: superoxide dismutase
CAT: catalase
GSTA2: glutathione S-transferase α2
PKA: protein kinase A
GCLC: glutamate-cysteine ligase catalytic subunit
ALT: alanine aminotransferase
AST: aspartate aminotransferase
CREA: creatinine
UREA: urea nitrogen
NO: nitric oxide
MDA: malondialdehyde
GSH: glutathione
ALP: lkaline phosphatase
ALB: albumin
IL-1β: interleukin-1β
cGAS: cyclic GMP-AMP synthase
STING: stimulators of interferon gene
